# Deep Infiltrating Endometriosis: Diagnostic Accuracy of Preoperative Magnetic Resonance Imaging with Respect to Morphological Criteria

**DOI:** 10.3390/diagnostics13101794

**Published:** 2023-05-19

**Authors:** Sebastian Harth, Fritz C. Roller, Felix Zeppernick, Ivo Meinhold-Heerlein, Gabriele A. Krombach

**Affiliations:** 1 Department of Diagnostic and Interventional Radiology, Justus-Liebig-University Giessen, Klinikstr. 33, 35392 Giessen, Germany; fritz.c.roller@radiol.med.uni-giessen.de (F.C.R.); gabriele.krombach@uniklinikum-giessen.de (G.A.K.); 2 Department of Gynecology and Obstetrics, Justus-Liebig-University Giessen, Klinikstr. 33, 35392 Giessen, Germany; felix.zeppernick@gyn.med.uni-giessen.de (F.Z.); ivo.meinhold-heerlein@gyn.med.uni-giessen.de (I.M.-H.)

**Keywords:** endometriosis, deep infiltrating endometriosis, magnetic resonance imaging, laparoscopy, predictive value of tests

## Abstract

Several current guidelines recommend imaging in the diagnostic work-up of deep infiltrating endometriosis (DIE). The purpose of this retrospective diagnostic test study was to evaluate the diagnostic accuracy of MRI compared to laparoscopy for the identification of pelvic DIE, considering lesion morphology using MRI. In all, 160 consecutive patients were included who received pelvic MRI for evaluation of endometriosis between October 2018 and December 2020 and underwent subsequent laparoscopy within 12 months of the MRI examination. MRI findings were categorized for suspected DIE using the Enzian classification and were additionally graded using a newly suggested deep infiltrating endometriosis morphology score (DEMS). Endometriosis was diagnosed in 108 patients (all types, i.e., purely superficial and DIE), of which 88 cases were diagnosed with DIE and 20 with solely superficial peritoneal endometriosis (i.e., not deep infiltrating endometriosis/DIE). The overall positive and negative predictive values of MRI for the diagnosis of DIE, including lesions with assumed low and medium certainty of DIE on MRI (DEMS 1–3), were 84.3% (95% CI: 75.3–90.4) and 67.8% (95% CI: 60.6–74.2), respectively, and 100.0% and 59.0% (95% CI: 54.6–63.3) when strict MRI diagnostic criteria were applied (DEMS 3). Overall sensitivity of MRI was 67.0% (95% CI: 56.2–76.7), specificity was 84.7% (95% CI: 74.3–92.1), accuracy was 75.0% (95% CI: 67.6–81.5), positive likelihood ratio (LR+) was 4.39 (95% CI: 2.50–7.71), negative likelihood ratio (LR-) was 0.39 (95% CI: 0.28–0.53), and Cohen’s kappa was 0.51 (95% CI: 0.38–0.64). When strict reporting criteria are applied, MRI can serve as a method to confirm clinically suspected DIE.

## 1. Introduction

Endometriosis is defined as the presence of endometrium-like epithelium and/or stroma outside the endometrium and myometrium, and deep infiltrating endometriosis (DIE) is characterized by the presence of endometrium-like tissue lesions, extending on or under the peritoneal surface [1]. It is estimated that about 10–15% of women of childbearing age are affected by endometriosis [2]. DIE is the most aggressive form of endometriosis, affecting approximately 20% of women who suffer from endometriosis [3].

Current guidelines recommend the use of imaging (ultrasound or MRI) in the diagnostic work-up for endometriosis [4,5] or recommend optional MRI in cases of suspected involvement of the bowel, urinary bladder, or ureter [6,7]. A sensitivity of 94% and a specificity of 77% of preoperative MRI for the detection of deep infiltrating endometriosis have been reported [8]. In the World Endometriosis Society consensus on the classification of endometriosis (2017), a weak consensus was reached on the recommendation of preoperative use of the Enzian classification [9].

The inclusion of morphological MRI criteria in the assessment of the accuracy of preoperative MRI has not previously been well established, although differences in morphology are known: DIE may manifest as pelvic nodules, plaque-like lesions, or adhesions. Frequently, the lesions have an irregular, spiculated shape; the signal is mostly T1w-intermediate and T2w-hypointense. Small T1w- or T2w-hyperintense foci are often detectable and correspond to endometrial glands [10].

The objectives of the present study were to evaluate the diagnostic accuracy of magnetic resonance imaging (MRI) for DIE in comparison with findings of surgery for different lesion morphologies on MRI and to develop a new classification which reflected the lesion morphology and, thus, the assumed certainty of MRI findings.

## 2. Materials and Methods

### 2.1. Characteristics of the Patients

**Figure 1:**
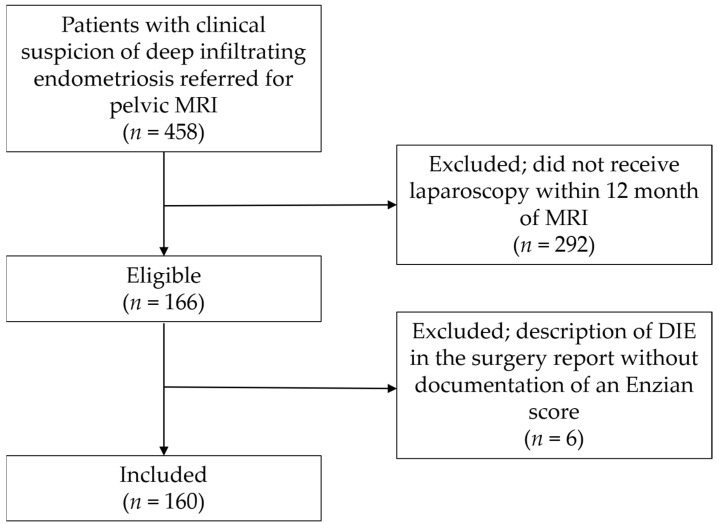
Patient recruitment flowchart. MRI, magnetic resonance imaging; DIE, deep infiltrating endometriosis.

All consecutive patients were retrospectively identified from a list of patients who underwent MRI for evaluation of pelvic endometriosis between October 2018 and December 2020 after clinical gynecological examination and transvaginal ultrasound (*n* = 458). Of these, 292 patients who had not undergone laparoscopy within 12 months of the MRI examination were excluded, and 6 patients with description of DIE in the surgery report without documentation of an Enzian score were excluded, leaving 160 patients for inclusion (Figure 1). Characteristics of the study population are presented in Table 1. No adverse events occurred during MRI examinations. The rationale for the chosen time interval between index test and reference test was the common assumption that, over a longer period of time, the disease status might have changed [8]. This study was approved by the institutional review board (IRB) (209/22).

MRI scans were performed using two 1.5-T magnet scanners (*n* = 92 and *n* = 66) and a 3-T scanner (*n* = 2). The MRI acquisition protocol included the sequences recommended in current guidelines and reviews [11,12,13]: T2-weighted FSE (fast spin echo) sequences in axial, sagittal, and coronal orientation; axial T1-weighted FSE sequences without and with fat suppression. In accordance with guidelines, the MRI examinations were scheduled without regard to the menstrual cycle [11]. Patient preparation included rectal opacification with water (*n* = 156) and vaginal opacification with ultrasound gel (*n* = 133). These measures are considered ‘optional’ or ‘conditionally recommended’ in current guidelines and are performed by many teams [11,14]. No further bowel preparation was carried out prior to the examination, which is in accordance with the guidelines of the Society of Abdominal Radiology [14] and is consistent with the practice of many teams [11]. To achieve moderate filling of the urinary bladder, as recommended [11,14], patients were asked to empty their bladder 1 h prior to the examination. As recommended in recent guidelines [14], an anti-peristaltic agent (IV hyoscine butylbromide 20 mg) and IV contrast agents were administered in most cases (gadoteridol 0.1 mmol/kg) (*n* = 158), including acquisition of axial and sagittal T1-weighted FSE sequences with fat suppression and T1-weighted gradient–echo urography.

### 2.2. Research Methods

**Figure 2:**
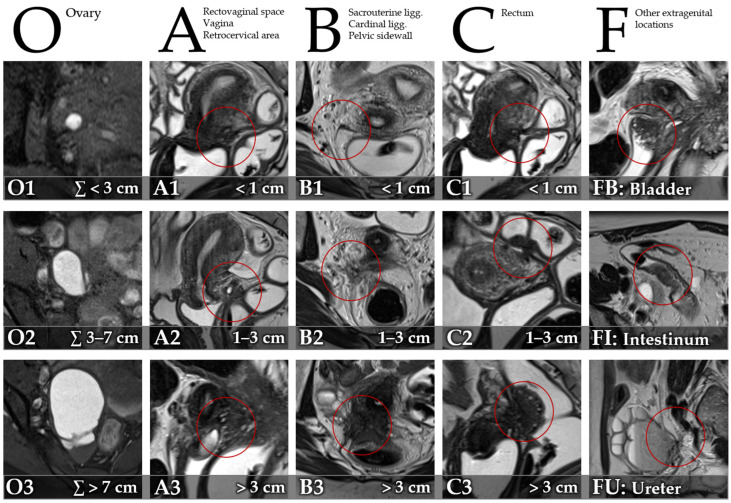
Description of the Enzian classification of endometriosis on MRI for compartments/organs O (T1-weighted FSE [fast spin echo] with fat suppression, axial), A (T2-weighted FSE, sagittal), B (T2-weighted FSE, axial), C (T2-weighted FSE, sagittal), FB (T2-weighted FSE, sagittal), FI (T2-weighted FSE, axial), and FU (T2-weighted FSE, sagittal), adapted from Keckstein et al. [15].

MRI images were reviewed on a Picture Archiving and Communication System (PACS) workstation. A radiologist with 7 years’ experience in pelvic MRI reviewed the images, blinded to results of surgery and histopathology. Clinical information was available to the reader of the index test. Presence and size of DIE were recorded utilizing the Enzian classification [15] (Figure 2) for compartments/organs A (rectovaginal space, vagina, retrocervical area), B (sacrouterine ligg., cardinal ligaments, pelvic sidewall), C (rectum), O (ovary), FB (bladder), FU (ureter), FI (intestinum), and FO (other). Size of lesions was graded for compartments A, B, and C (1: <1 cm, 2: 1–3 cm, 3: >3 cm). The summed size of endometriomas was graded for category O (1: ∑ < 3 cm, 2: ∑ 3–7 cm, 3: ∑ > 7 cm).

**Figure 3:**
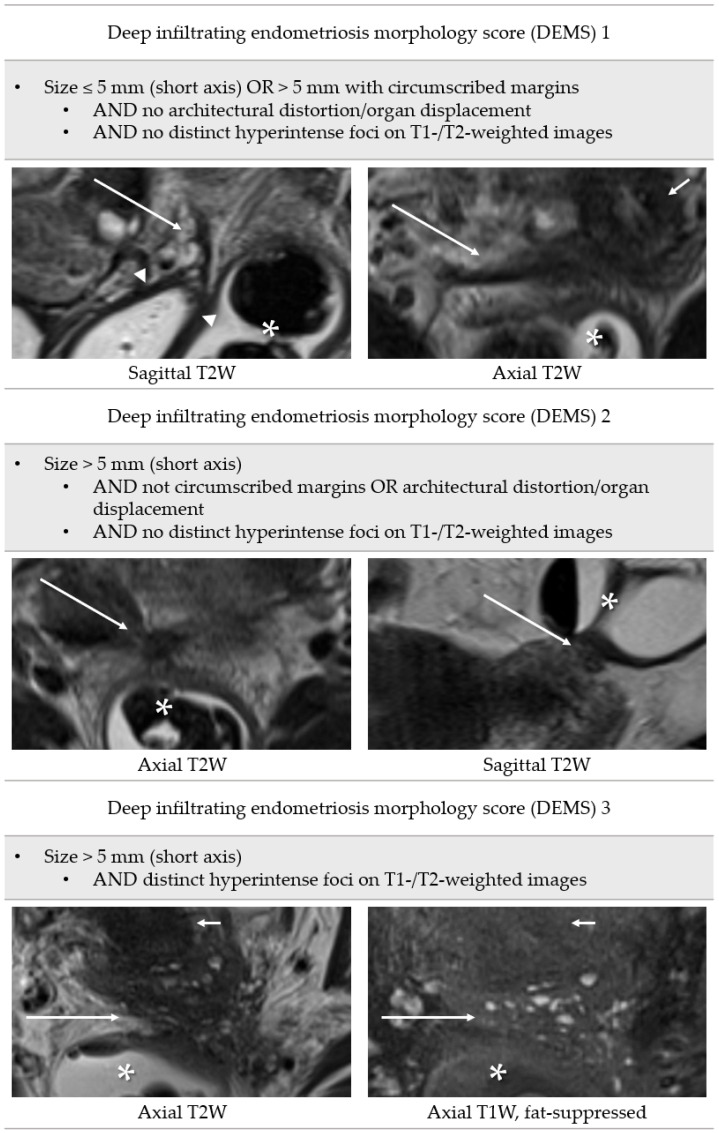
Definition of deep infiltrating endometriosis morphology score. Long arrows: deep infiltrating endometriosis; arrowheads: vaginal vault; asterisks: rectosigmoid; short arrows: cervix uteri.

A scale that incorporates the lesion morphology was developed for MRI and named deep infiltrating endometriosis morphology score (DEMS). This score provides a clear definition of positive findings of the index test and includes assessment of the following features in predominantly T2-hypointense nodules or thickened uterine ligaments: size of lesion, measured in short axis for all lesions (≤5 mm, >5 mm); margins (circumscribed, not circumscribed); architectural distortion/organ displacement; and distinct hyperintense foci on T1-weighted or T2-weighted images (Figure 3).

If different DIE-suspicious findings were present in a patient’s MRI, the highest DEMS determined was noted. Diagnosis of ovarian endometrioma was made according to previously defined criteria [16].

Operations were performed by experienced senior surgeons at a training center for gynecological minimally invasive surgery, certified by the Working Group for Gynecological Endoscopy of the German Society for Gynecology and Obstetrics and by EuroEndoCert (EuroEndoCert GmbH, Mannheim, Germany), on behalf of the Scientific Endometriosis Foundation (Stiftung Endometriose-Forschung, Westerstede, Germany). Results of the index test (MRI) were available to the surgeons. Enzian scores were noted as given in the surgery reports, and additional findings as well as results of histopathology were recorded. Description of DIE with documentation using the Enzian classification in the surgical report was considered a positive reference test. Cases in which no DIE was described and, accordingly, no positive Enzian classification was given were counted as having a negative reference test. As mentioned above, patients with a DIE description in the surgery report without documentation of an Enzian score had been excluded from the study cohort (*n* = 6). In 10 cases, DIE was described in the surgery report and an Enzian score was given for at least one of the DIE compartments/organs (A, B, C, FB, FU, FI, FO), but the diagnosis could not be clearly confirmed histopathologically. These cases were considered to have a positive reference test in accordance with the current ESHRE guideline: Endometriosis (2022), which recommends that laparoscopic identification of endometriotic lesions be confirmed by histology, although negative histology does not rule out the disease [4]. In 10 other cases, no DIE, but only superficial peritoneal endometriosis, was described in the surgery report and peritoneal endometriosis was confirmed histopathologically. These cases were considered to have a negative reference test, as no DIE was diagnosed.

### 2.3. Methods for Statistical Analysis

Statistical analyses were performed using IBM SPSS Statistics 29.0 (IBM, Armonk, New York, NY, USA) and MedCalc 20.215 (MedCalc Software Ltd., Ostend, Belgium). A sample size estimation was performed, following the reported overall sensitivity of 94% and specificity of 77% of preoperative MRI [8] and assuming a disease prevalence of approximately 50% in our cohort of symptomatic patients [17,18]. Accordingly, the estimated sample size was 136 for DIE diagnosis overall (marginal error 0.10 with a confidence level of 95%, type I error 5%) [19,20]. Sensitivity, specificity, positive and negative predictive values (PPV/NPV), positive and negative likelihood ratios, accuracy, and agreement (Cohen’s kappa coefficient, κ) were calculated for dichotomous data of the category ‘DIE overall’ and the Enzian compartments A, B, and C. The analysis was carried out for different DEMS values: with all MR-positive cases (DEMS 1–3) considered, with only DEMS 2–3 cases rated as MR-positive (i.e., DEMS 0–1 rated as MR-negative), and with only DEMS 3 cases rated as MR-positive (i.e., DEMS 0–2 rated as MR-negative). Clopper–Pearson confidence intervals were calculated for sensitivity, specificity, and accuracy. Confidence intervals for likelihood ratios were calculated using the log method. The standard logit method was used for the estimation of the confidence intervals of the predictive values (PPV/NPV). McNemar’s exact test was used to compare sensitivities and specificities of dichotomous MRI categorizations for Enzian compartments A, B, and C and category ‘DIE overall’ for different DEMS values. To compare sensitivities, McNemar’s test was used among patients with positive reference standard alone, while to compare specificities, McNemar’s test was applied among patients with negative reference standard alone [21]. Confidence intervals of the differences of sensitivities and specificities were calculated using the method proposed by Newcombe [22]. Kendall Tau-b values were calculated to assess the association of the Enzian classification subcategories for compartments A, B, and C (0–3) between preoperative MRI and surgical findings, independent of DEMS level (DEMS 0–3). Dichotomous data for DIE diagnosis overall (DIE+/DIE−) and the Enzian compartments/organs A, B, C, FB, FU, FI, and FO were tabulated, layered by DEMS. Cohen’s kappa coefficients (κ) were calculated as measure of agreement, independent of DEMS (DEMS 0–3). Kendall Tau-c was calculated as a measure of agreement between MRI (O0–3) and surgery (O+/O−) for the detection of endometriomas. An interobserver test was performed. To this end, MRI examinations of 20 patients, drawn at random from within the samples, were assessed by a second radiologist with 5 years’ experience in pelvic MRI in a blind examination. Cohen’s kappa coefficients (κ) were calculated for dichotomous variables. Quadratic weighted kappa coefficients (κ_w_) were calculated for ordinal scaled variables. Values between 0.81 and 1.00 were considered excellent (‘almost perfect’) agreement, 0.61–0.80 were substantial agreement, 0.41–0.60 were moderate agreement, 0.21–0.40 were fair agreement, and 0.00–0.20 were slight agreement [23].

## 3. Results

Endometriosis (superficial peritoneal endometriosis and DIE) was diagnosed intraoperatively in a total of 108/160 (67.5%) patients. Of these, 88/160 (55.5%) patients were diagnosed with DIE and counted as having a positive reference test and 20/160 (12.5%) patients were diagnosed with only superficial peritoneal endometriosis. The diagnosis of endometriosis was confirmed histopathologically in 88/160 cases (55.0%). Of these, 10/88 (11.4%) cases related to patients with only superficial peritoneal endometriosis, and 78/88 (88.6%) related to patients with DIE. Additionally, 10/88 (11.4%) cases of surgically confirmed DIE could not be clearly confirmed histopathologically. As stated above, these cases were considered to have a positive reference test, in accordance with current guidelines [4]. Endometriomas were found surgically in 39/160 (24.4%) cases, of which only one patient had an isolated endometrioma without superficial or deep infiltrating endometriosis. In the 52/160 (32.5%) patients without intraoperative diagnosis of endometriosis, alternative diagnoses were as follows (several diagnoses possible): adhesions (*n* = 24), uterine fibroids (*n* = 7), adenomyosis uteri (*n* = 6), ovarian teratoma (*n* = 2), hemorrhagic ovarian follicular cyst (*n* = 1), benign simple ovarian cyst (*n* = 1), ovarian serous cystadenoma (*n* = 1), pelvic inflammatory disease (*n* = 1), pelvic venous congestion (*n* = 1), peritoneal pseudocysts (*n* = 1), and low-grade appendiceal mucinous neoplasm (*n* = 1). In 12/160 (7.5%) patients, laparoscopy was unremarkable.

### 3.1. Performance of MRI for Diagnosis of DIE

**Figure 4:**
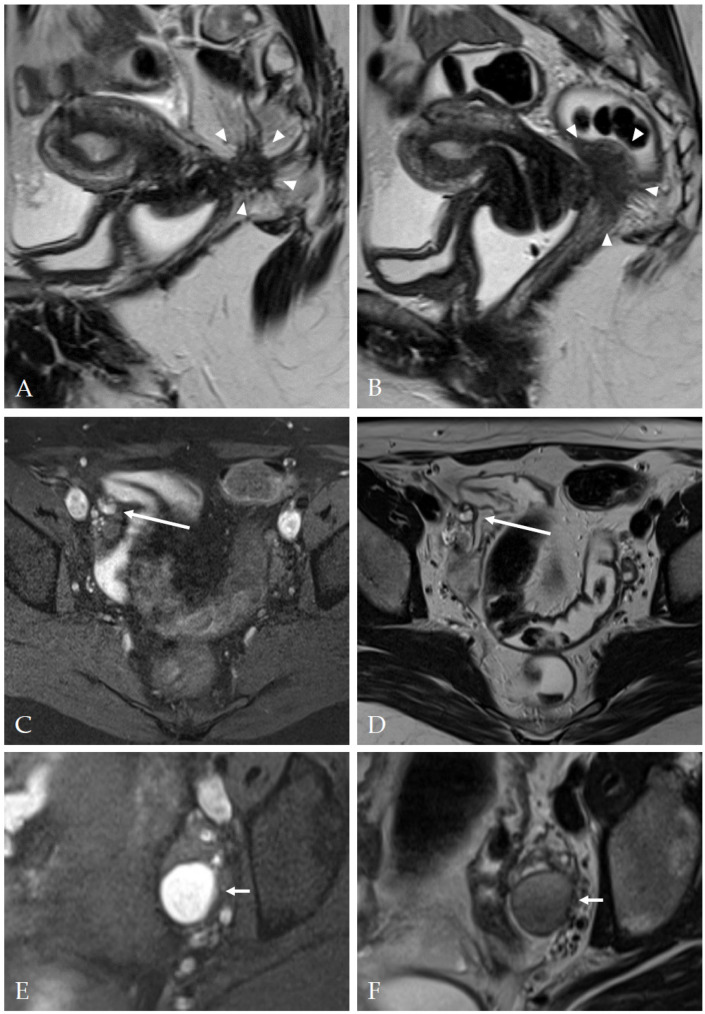
Magnetic resonance imaging examples of deep infiltrating endometriosis and endometriomas. (**A**,**B**) Two slices of a sagittal T2-weighted FSE (fast spin echo) sequence, depicting a mostly hypointense mass with ill-defined margins and hyperintense foci (arrowheads) invading the vaginal vault and the rectum (Enzian A2, B3, C3, DEMS (deep infiltrating endometriosis morphology score) 3). (**C**) Axial T1 FSE with fat suppression and (**D**) axial T2 FSE sequence showing a nodule with T1- and T2-hyperintense foci at the right pelvic sidewall (long arrow) (Enzian B1, DEMS 3). (**E**) Axial T1 FSE with fat suppression and (**F**) axial T2 TSE showing a typical endometrioma (short arrows, T1-hyperintense, T2-hypointense with ‘shading’) (Enzian O2).

The overall accuracy of MRI for diagnosis of DIE was 75.0 (95% CI: 67.6–81.5). Examples of typical MRI findings are shown in Figure 4.

The sensitivity, specificity, positive and negative predictive values, positive and negative likelihood ratios, accuracy, and Cohen’s kappa coefficients (κ) for DIE diagnosis overall (DIE+/DIE−) and for Enzian compartments A, B, and C (dichotomized), layered by DEMS, are shown in Table 2. Enzian locations FB, FU, FI, and FO were excluded from this analysis due to the low number of MR-positive findings.

Table 3 depicts the agreement of the Enzian classification subcategories of compartments A, B, and C (0–3) of preoperative MRI and surgery, layered by DEMS, whilst also giving Kendall Tau-b values, independent of DEMS (DEMS 0–3).

Table 4 and Table 5 show agreement of preoperative MRI and surgery for DIE diagnosis overall (DIE+/DIE−) and for Enzian compartments A, B, and C (dichotomized), as well as locations FB, FU, FI, and FO, layered by DEMS level, as well as Cohen’s kappa coefficients (κ), independent of DEMS (DEMS 0–3). The findings of the Enzian category FI (intestinum) were localized in the ileum (*n* = 2) and the sigmoid colon (*n* = 10). The findings of the Enzian category FO (other) were localized at the peritoneum outside Enzian compartments A, B, and C (*n* = 4) and at the lumbosacral plexus (*n* = 1).

### 3.2. Performance of MRI for Diagnosis of Endometriomas

For ovarian endometriomas, the preoperative classification of the Enzian score O0–3 was compared to the dichotomized surgical findings (O+/O−), since an exact assignment to the levels of the Enzian classification for endometriomas was not consistently available. The MRI classification O0 was assigned 103 times, and, in 101 of these cases, no endometrioma was described surgically, while in 2 cases, an endometrioma was described. The MRI classification O1 was assigned 17 times, and, in 10 of these cases, no endometrioma was described surgically, while in 7 cases, an endometrioma was described. The MRI classification O2 was assigned 29 times, and, in 8 of these cases, no endometrioma was described surgically, while in 21 cases, an endometrioma was described. MRI classification O3 was assigned 11 times, and, in 2 of these cases, no endometrioma was described surgically, while in 9 cases, an endometrioma was described. Kendall Tau-c as a measure of agreement between MRI (O0–3) and surgery (O+/O−) for the detection of endometriomas was 0.62 (95% CI: 0.48–0.75). Further statistical parameters were as follows: sensitivity 94.9% (95% CI: 82.7–99.4%), specificity 83.5% (95% CI: 75.6–89.6%), positive predictive value (PPV) 64.9% (95% CI: 55.2–73.5%), negative predictive value (NPV) 98.1% (95% CI: 92.9–99.5%), and accuracy 86.3% (95% CI: 79.9–91.2%).

### 3.3. Influence of Deep Infiltrating Endometriosis Morphology Score

Table 6 depicts differences in sensitivities and specificities for different DEMS values with indication of 95% confidence intervals and *p*-values of the results of the McNemar’s exact test.

### 3.4. Interobserver Test

Results of the interobserver test are shown in Table 7.

## 4. Discussion

Our investigation yielded specificities in the range of 82.5% to 98.5% and sensitivities in the range from 40.0% to 82.1% for the preoperative MRI-based application of the Enzian classification for compartments A, B, and C. These results emphasize the role of preoperative MRI as a method to confirm the diagnosis of DIE and reaffirm that a negative MRI cannot exclude endometriosis, due to possible false-negative findings of MRI. Increases in specificity of DIE diagnosis overall could be observed with stricter morphological criteria for Enzian compartments A and B (i.e., higher DEMS). As to be expected, the gain in specificity was at the cost of sensitivity, which decreased in almost all compartments with increasing DEMS. When considering accuracy, Cohen’s kappa, and Youden’s index, the best agreement between preoperative MRI and surgery in our study could be shown for ‘DIE overall, DEMS 2’3′, ‘A, DEMS 3’, ‘B, DEMS 2–3’ and ‘C, DEMS 2–3’, with sensitivities of 59.1%, 55.3%, 55.0%, and 82.1% and specificities of 94.4%, 96.5%, 88.8%, and 98.5%, respectively. Hence, the inclusion of morphological criteria in the diagnosis can help to assess the predictive value of MRI findings more accurately and to communicate findings clearly between radiology and gynecology.

### 4.1. Strengths and Limitations

The strengths of our study are adequate sample size, inclusion of consecutive patients, not excluding patients with prior surgeries or patients taking certain hormone medications, including patients independent of the results of the reference test, limited time difference between index and reference test, and blinded analysis of images.

The limitations are the retrospective design and the conduction in a single tertiary care center. Laparoscopy served as the reference standard in our study, although its limitations are known, including limitations in the identification of deep endometriotic lesions that are hidden by adhesions and inflammation, as well as limitations in the prediction of the depth of invasion of rectosigmoid lesions [24]. Goncalves et al. have shown in their study of 120 patients that diagnostic laparoscopy was able to detect retrocervical, ovarian, and bladder endometriosis with similar sensitivity and specificity as transvaginal ultrasound with bowel preparation, whereas for vaginal and rectosigmoid endometriosis, diagnostic laparoscopy had lower sensitivity and specificity [24].

The extent to which patient preparation and usage of IV contrast agents may have influenced the results of our study remains unanswered. Patient preparation in our cohort included rectal and vaginal opacification in most cases, and IV contrast agents were applied in most patients. There is currently no international consensus regarding patient preparation and IV contrast agents, and current guidelines of the European society of urogenital radiology (ESUR) consider vaginal and rectal opacification, as well as the use of gadolinium, as ‘options’ in the evaluation of DIE [11].

### 4.2. Deep Endometriosis Morphology Score (DEMS)

The results for different DEMS values show that the morphology of lesions plays a subordinate role for compartment C (rectum) with regard to the certainty of the diagnosis of DIE; thus, it seems to be useful in practice to also report smaller lesions on the rectum and lesions without typical hyperintense foci (DEMS 1/2). For compartments A and B, attention to the morphology of the lesions can apparently provide guidance in clinical practice: In compartment A, there is no significant loss of sensitivity if lesions of minor conspicuity (DEMS 1) are not reported, but an improvement in specificity can be achieved; In compartment B, however, the loss of sensitivity is significant if lesions of minor conspicuity (DEMS 1) are not reported. On the other hand, if the morphology of lesions in compartment B is typical (DEMS 3: >5 mm transverse diameter, hyperintense foci), the diagnosis of DIE can be considered very certain.

We found superior results for Enzian compartment C compared to compartments A and B in several respects (agreement of MRI and surgery with and without dichotomisation; interobserver variability). This is consistent with the observations of other authors, that assignment of DIE lesions to compartments A and B can be difficult, especially when lesions are located at the border between two overlapping compartments [25]. In addition, it can be challenging to accurately determine the size of lesions on MRI due to the indistinct borders and streaky extensions of the lesions. We have, therefore, proposed a measurement of lesions in the short axis for DEMS classification, assuming a better indication of the likelihood of DIE. In addition, possible false-negative results of the reference standard have to be considered, as discussed above [24].

The agreement of the Enzian classification subcategories of compartments A, B, and C (0–3) of preoperative MRI and surgery were moderate for compartments A and B, and strong for compartment C. It can therefore be assumed that subclassification in compartments A, B, and especially C is accompanied by a further increase in information compared to the corresponding dichotomous variables.

### 4.3. Enzian A, B, C: Previous Studies

In a recent prospective multicenter study by Enzelsberger et al. [18], preoperative use of the Enzian classification was assessed. Sensitivities of 79%, 68%,and 79% and specificities of 88%, 82%, and 92% of preoperative MRI were reported for compartments A, B, and C of the Enzian classification, respectively (*n* = 168), in a cohort with a similar proportion of positive reference standard (53.1%) in the whole sample (*n* = 1062) compared to our study (55.0%).

The retrospective study of Burla et al. [26] yielded sensitivities of 95.2%, 78.4%, and 91.4% and specificities of 95.7%, 100.0%, and 91.4% for Enzian compartments A, B, and C, respectively, while including only patients (*n* = 63) with surgical DIE verification. Thus, the comparability to our results is limited.

Thomassin-Naggara et al. [27] reported concordance of MRI-based and surgical Enzian classifications in compartments A, B, and C of 78.7%, 34.7%, and 82.7%, respectively (*n* = 150), although specific statistical parameters (e.g., sensitivities, specificities) were stated for several anatomical regions, but not explicitly for the compartments of the Enzian classification.

Sensitivities of 98%, 97%, and 86% and specificities of 94%, 99%, and 98% were reported by Di Paola et al. [28] for Enzian compartments A, B, and C, respectively. However, only patients with histopathological results of laparoscopic or surgical therapy were included, and a higher positive rate of the reference standard of 71.3% was depicted.

Although patients with certain prior surgeries were excluded and the prevalence of DIE in the study cohort was 100%, Hernández Gutiérrez et al. [29] reported lower sensitivities of 74%, 33%, 67%, and 69% and specificities of 64%, 93%, 43%, and 87% for preoperative MRI-based assessment of compartments/organs ‘recto-vaginal space’, ‘vagina’, ‘utero-sacral ligaments’, and ‘recto-sigmoid’, respectively.

### 4.4. Enzian FB, FU, FI, FO: Predictive Value of MRI

We found substantial agreement between preoperative MRI and surgery for the whole sample (DEMS 0–3) for Enzian categories FI (κ: 0.75; 95% CI: 0.53–0.96) and FU (κ: 0.80; 95% CI: 0.41–1) and moderate agreement for Enzian categories FB (κ: 0.55; 95% CI: 0.23–0.87) and FO (κ: 0.59; 95% CI: 0.22–0.96). However, the numbers in these locations were too small for a final judgement to be made. The results of the interobserver test indicate a certain degree of agreement between the categories FB, FI, and FO, although the confidence intervals are wide due to the small sample sizes in these categories. Burla et al. [30] reported substantial interobserver agreement for category FB (κ: 0.64), slight agreement for category FI (κ: 0.20), and poor agreement for category FO (κ: −0.03), Manganaro et al. [31] found substantial interobserver agreement (κ: 0.63) for the category ‘extragenital deep infiltrating endometriosis (F)’, and Thomassin-Naggara et al. [27] reported excellent interobserver agreement for the category ‘bladder’ (κ: 0.9).

### 4.5. Endometriomas: Predictive Value of MRI

Agreement between MRI (O0–3) and surgery (O+/O−) for the detection of endometriomas was substantial, with sensitivity of 94.9% and specificity of 83.5%, similar to the results of other authors: Thomassin-Naggara et al. reported a sensitivity of 86% and a specificity of 80% for a senior reader [27]. The meta-analysis of three studies in the review of Nisenblat et al. revealed summary sensitivity and specificity of 0.95 (95% CI: 0.90–1.00) and 0.91 (95% CI: 0.86–0.97) of MRI for ovarian endometriosis [8].

## 5. Conclusions

The results of our study emphasize the role of preoperative MRI as a method to confirm the diagnosis of DIE and reaffirm that a negative MRI cannot exclude endometriosis due to possible false-negative findings on MRI. The inclusion of morphological criteria in the diagnosis in the form of the deep infiltrating endometriosis morphology score (DEMS) can help to assess the predictive value of MRI findings more accurately and to communicate findings clearly between radiology and gynecology. Further studies based on larger study populations are desirable for external validation of the presented morphological score and confirmation of our results, as well as to clarify the optimal preparation protocol and the value of IV contrast administration for MRI in suspected endometriosis.

## Figures and Tables

**Table 1:** Demographics and baseline characteristics.

Characteristic	*N*/Total (%, Unless Shown Otherwise)
Age (years) mean ± SD, range	33.0 ± 7.2, 19–55
Time interval from MRI to surgery (days) Mean ± SD, range	75 ± 66, 1–337
Prior abdominal surgery	85/160 (53)
Prior caesarean section	20/160 (13)
Clinical symptoms	
Chronic pelvic pain	138/160 (86)
Dysmenorrhea	87/160 (54)
Dyspareunia	47/160 (29)
Infertility	38/160 (24)
Constipation/diarrhoea	11/160 (7)
Dyschezia	28/160 (18)
Dysuria	20/160 (13)
Abnormal uterine bleeding	19/160 (12)
Type of surgery	
Hysterectomy	29/160 (18)
Partial colpectomy	9/160 (6)
Discoid resection	2/160 (1)
Rectal shaving	12/160 (8)
Colorectal resection	10/160 (6)

MRI, magnetic resonance imaging.

**Table 2:** Statistical parameters of agreement of preoperative MRI and surgery for DIE diagnosis overall (DIE+/DIE−) and for Enzian compartments A, B, and C (dichotomized), layered by DEMS.

DEMS	MRI	MR+ *N*/Total (%)	Se. 95% CI (%)	Sp. 95% CI (%)	PPV 95% CI (%)	NPV 95% CI (%)	LR+ 95% CI	LR− 95% CI	Accuracy 95% CI (%)	Cohen’s Kappa (κ) 95% CI	YI
MR−: 0 MR+: 1–3	DIE	70/160 (43.8)	67.0 (56.2–76.7)	84.7 (74.3–92.1)	84.3 (75.3–90.4)	67.8 (60.6–74.2)	4.39 (2.50–7.71)	0.39 (0.28–0.53)	75.0 (67.6–81.5)	0.51 (0.38–0.64)	0.52
	A	40/160 (25.0)	57.5 (42.2–71.7)	88.5 (81.1–93.7)	67.5 (54.1–78.6)	83.3 (78.1–87.5)	4.99 (2.83–8.81)	0.48 (0.34–0.67)	79.4 (72.3–85.4)	0.48 (0.33–0.63)	0.46
	B	63/160 (39.4)	61.3 (49.7–71.9)	82.5 (72.4–90.1)	77.8 (67.8–85.3)	68.0 (61.4–74.1)	3.50 (2.11–5.81)	0.47 (0.35–0.63)	71.9 (64.2–78.7)	0.44 (0.30–0.57)	0.44
	C	26/160 (16.3)	82.1 (63.1–93.9)	97.7 (93.5–99.5)	88.5 (71.2–96.0)	96.3 (92.1–98.3)	36.14 (11.65–112.10)	0.18 (0.08–0.40)	95.0 (90.4–97.8)	0.82 (0.70–0.94)	0.80
MR−: 0–1 MR+: 2–3	DIE	56/160 (35.0)	59.1 (48.1–69.5)	94.4 (86.4–98.5)	92.9 (83.2–97.2)	65.4 (59.4–71.0)	10.64 (4.04–28.01)	0.43 (0.33–0.56)	75.0 (67.6–81.5)	0.52 (0.40–0.63)	0.54
	A	34/160 (21.3)	57.5 (42.2–71.7)	93.8 (87.7–97.5)	79.4 (64.4–89.2)	84.1 (79.1–88.1)	9.27 (4.34–19.80)	0.45 (0.33–0.63)	83.1 (76.4–88.6)	0.56 (0.41–0.70)	0.51
	B	53/160 (33.1)	55.0 (43.5–66.2)	88.8 (79.7–94.7)	83.0 (71.9–90.3)	66.4 (60.5–71.8)	4.89 (2.56–9.33)	0.51 (0.39–0.65)	71.9 (64.2–78.7)	0.44 (0.31–0.57)	0.44
	C	25/160 (15.6)	82.1 (63.1–93.9)	98.5 (94.6–99.8)	92.0 (74.2–97.9)	96.3 (92.1–98.3)	54.21 (13.55–216.83)	0.18 (0.08–0.40)	95.6 (91.2–98.2)	0.84 (0.73–0.96)	0.81
MR−: 0–2 MR+: 3	DIE	38/160 (23.8)	43.2 (32.7–54.2)	100.0 (95.0–100.0)	100.0	59.0 (54.6–63.3)	-	0.57 (0.47–0.68)	68.8 (61.0–75.8)	0.41 (0.30–0.52)	0.43
	A	30/160 (18.8)	55.3 (40.1–69.8)	96.5 (91.2–99.0)	86.7 (70.6–94.6)	83.9 (79.0–87.7)	15.63 (5.77–42.32)	0.46 (0.34–0.64)	84.4 (77.8–89.6)	0.58 (0.44–0.72)	0.52
	B	36/160 (22.5)	40.0 (29.2–51.6)	95.0 (87.7–98.6)	88.9 (74.8–95.6)	61.3 (56.8–65.6)	8.00 (2.97–21.58)	0.63 (0.52–0.76)	67.5 (59.7–74.7)	0.35 (0.23–0.47)	0.35
	C	24/160 (15.0)	78.6 (59.1–91.7)	98.5 (94.6–99.8)	91.7 (73.3–97.8)	95.6 (91.4–97.8)	51.86 (12.93–207.98)	0.22 (0.11–0.44)	95.0 (90.4–97.8)	0.82 (0.69–0.94)	0.77

MRI, magnetic resonance imaging; DIE, deep infiltrating endometriosis; DEMS, deep infiltrating endometriosis morphology score; MR+, MRI positive; Se., Sensitivity; Sp., Specificity; LR+, positive likelihood ratio; LR−, negative likelihood ratio; YI, Youden’s index; MR−, MRI negative definition; MR+, MRI positive definition.

**Table 3:** Agreement of Enzian scores of compartments A, B, and C of preoperative MRI and surgery, layered by DEMS.

		Surgery					Surgery					Surgery			
DEMS	MRI	A0	A1	A2	A3	MRI	B0	B1	B2	B3	MRI	C0	C1	C2	C3
0	A0	80	9	1	0	B0	62	15	10	3	C0	87	3	0	0
	A1	0	0	0	0	B1	0	0	0	0	C1	0	0	0	0
	A2	0	0	0	0	B2	0	0	0	0	C2	0	0	0	0
	A3	0	0	0	0	B3	0	0	0	0	C3	0	0	0	0
1	A0	7	1	0	0	B0	2	0	2	0	C0	12	1	0	0
	A1	6	0	0	0	B1	3	0	1	0	C1	1	0	0	0
	A2	0	0	0	0	B2	1	0	2	1	C2	0	0	0	0
	A3	0	0	0	0	B3	1	0	1	0	C3	0	0	0	0
2	A0	11	1	1	1	B0	1	0	0	0	C0	16	1	0	0
	A1	2	0	0	0	B1	0	2	0	1	C1	0	0	1	0
	A2	1	0	1	0	B2	1	1	4	1	C2	0	0	0	0
	A3	0	0	0	0	B3	4	2	1	0	C3	0	0	0	0
3	A0	2	1	3	2	B0	1	0	1	0	C0	14	0	0	0
	A1	0	2	4	1	B1	0	0	0	0	C1	0	1	0	0
	A2	4	1	4	8	B2	2	0	4	6	C2	2	5	1	0
	A3	0	2	2	2	B3	2	0	13	9	C3	0	3	2	10
0–3	A0 *	100	12	5	3	B0 ^†^	66	15	13	3	C0 ^‡^	129	5	0	0
	A1 *	8	2	4	1	B1 ^†^	3	2	1	1	C1 ^‡^	1	1	1	0
	A2 *	5	1	5	8	B2 ^†^	4	1	10	8	C2 ^‡^	2	5	1	0
	A3 *	0	2	2	2	B3 ^†^	7	2	15	9	C3 ^‡^	0	3	2	10

MRI, magnetic resonance imaging; DEMS, deep infiltrating endometriosis morphology score. * Kendall Tau-b (DEMS 0–3): 0.51 (95% CI: 0.37–0.64). ^†^ Kendall Tau-b (DEMS 0–3): 0.46 (95% CI: 0.35–0.58). ^‡^ Kendall Tau-b (DEMS 0–3): 0.83 (95% CI: 0.73–0.93).

**Table 4:** Agreement of preoperative MRI and surgery for DIE diagnosis overall (DIE+/DIE−) and for Enzian compartments A, B, and C (dichotomized), layered by DEMS.

		Surgery			Surgery			Surgery			Surgery	
DEMS	MRI	DIE−	DIE+	MRI	A−	A+	MRI	B−	B+	MRI	C−	C+
0	DIE−	61	29	A−	80	10	B−	62	28	C−	87	3
	DIE+	0	0	A+	0	0	B+	0	0	C+	0	0
1	DIE−	0	0	A−	7	1	B−	2	2	C−	12	1
	DIE+	7	7	A+	6	0	B+	5	5	C+	1	0
2	DIE−	0	0	A−	11	3	B−	1	0	C−	16	1
	DIE+	4	14	A+	3	1	B+	5	12	C+	0	1
3	DIE−	0	0	A−	2	6	B−	1	1	C−	14	0
	DIE+	0	38	A+	4	26	B+	4	32	C+	2	22
0–3	DIE− *	61	29	A− ^†^	100	20	B− ^‡^	66	31	C− ^§^	129	5
	DIE+ *	11	59	A+ ^†^	13	27	B+ ^‡^	14	49	C+ ^§^	3	23

MRI, magnetic resonance imaging; DIE, deep infiltrating endometriosis; DEMS, deep infiltrating endometriosis morphology score. * Kappa (κ) (DEMS 0–3): 0.51 (95% CI: 0.38–0.64). ^†^ Kappa (κ) (DEMS 0–3): 0.48 (95% CI: 0.33–0.63). ^‡^ Kappa (κ) (DEMS 0–3): 0.44 (95% CI: 0.30–0.57). ^§^ Kappa (κ) (DEMS 0–3): 0.82 (95% CI: 0.70–0.94).

**Table 5:** Agreement of preoperative MRI and surgery for Enzian locations FB, FU, FI, and FO, layered by DEMS.

		Surgery			Surgery			Surgery			Surgery	
DEMS	MRI	FB0 *	FB1 *	MRI	FU0 ^†^	FU1 ^†^	MRI	FI0 ^‡^	FI1 ^‡^	MRI	FO0 ^§^	FO1 ^§^
0	FB0	90	0	FU0	90	0	FI0	90	0	FO0	90	0
	FB1	0	0	FU1	0	0	FI1	0	0	FO1	0	0
1	FB0	12	1	FU0	14	0	FI0	12	0	FO0	13	0
	FB1	1	0	FU1	0	0	FI1	1	1	FO1	0	1
2	FB0	15	0	FU0	18	0	FI0	17	0	FO0	14	2
	FB1	2	1	FU1	0	0	FI1	1	0	FO1	1	1
3	FB0	33	1	FU0	35	0	FI0	28	1	FO0	36	0
	FB1	1	3	FU1	1	2	FI1	2	7	FO1	1	1
0–3	FB0	150	2	FU0	157	0	FI0	147	1	FO0	153	2
	FB1	4	4	FU1	1	2	FI1	4	8	FO1	2	3

MRI, magnetic resonance imaging; DIE, deep infiltrating endometriosis; DEMS, deep infiltrating endometriosis morphology score. * Kappa (κ) (DEMS 0–3): 0.55 (95% CI: 0.23–0.87). ^†^ Kappa (κ) (DEMS 0–3): 0.80 (95% CI: 0.41–1.00). ^‡^ Kappa (κ) (DEMS 0–3): 0.75 (95% CI: 0.53–0.96). ^§^ Kappa (κ) (DEMS 0–3): 0.59 (95% CI: 0.22–0.96).

**Table 6:** Differences of sensitivities and specificities for DIE overall and Enzian compartments A, B, and C (dichotomized) for different DEMS; *p*-values of McNemar’s exact test.

Category	DEMS A	DEMS B	Difference in Sensitivity (B-A), 95% CI	*p*-Value	Difference in Specificity (B–A), 95% CI	*p*-Value
DIE	1–3	2–3	−7.95 (−13.97 to −1.88)	0.016	9.72 (2.33 to 18.63)	0.016
	1–3	3	−23.86 (−32.44 to −14.42)	<0.001	15.28 (7.02 to 25.32)	<0.001
	2–3	3	−15.91 (−23.45 to −7.86)	<0.001	5.56 (−0.53 to 13.43)	0.125
A	1–3	2–3	0.00 (−4.16 to 4.16)	1.000	5.31 (0.69 to 10.96)	0.031
	1–3	3	−2.13 (−7.83 to 3.62)	1.000	7.96 (2.80 to 14.46)	0.004
	2–3	3	−2.13 (−7.83 to 3.62)	1.000	2.65 (−1.33 to 7.59)	0.250
B	1–3	2–3	−6.25 (−11.95 to 0.44)	0.063	6.25 (0.16 to 13.25)	0.063
	1–3	3	−21.25 (−29.92 to −11.74)	<0.001	12.50 (5.00 to 21.41)	0.002
	2–3	3	−15.00 (−22.76 to −6.73)	<0.001	6.25 (0.16 to 13.77)	0.063
C	1–3	2–3	0.00 (−10.16 to 10.16)	1.000	0.76 (−2.41 to 4.40)	1.000
	1–3	3	−3.57 (−15.65 to 7.84)	1.000	0.76 (−2.41 to 4.40)	1.000
	2–3	3	−3.57 (−15.65 to 7.84)	1.000	0.00 (−3.11 to 3.11)	1.000

DIE, deep infiltrating endometriosis; DEMS, deep infiltrating endometriosis morphology score.

**Table 7:** Results of the interobserver test (*n* = 20) for preoperative MRI categorization of DIE.

Category	R1 *N*	R2 *N*	Kappa (κ)/Weighted Kappa (κ_w_)
O0–3	O1: 5, O2: 5, O3: 1	O1: 3, O2: 3, O3: 2	κ_w_: 0.88 (95% CI: 0.76–0.99)
A0–3	A1:1, A2: 2, A3: 1	A1: 1, A2: 3, A3: 0	κ_w_: 0.57 (95% CI: 0.13–1.00)
B0–3	B1: 3, B2: 3, B3: 0	B1: 3, B2: 3, B3: 3	κ_w_: 0.44 (95% CI: 0.11–0.76)
C0–3	C1: 0, C2: 4, C3: 0	C1: 0, C2: 3, C3: 1	κ_w_: 0.89 (95% CI: 0.75–1.00)
DEMS	1: 3, 2: 3, 3: 5	I: 3, 2: 3, 3: 4	κ_w_: 0.75 (95% CI: 0.52–0.98)
FB	2	1	κ: 0.64 (95% CI: 0.19–1.00)
FU	1	0	-
FI	2	3	κ: 0.77 (95% CI: 0.35–1.00)
FO	2	1	κ: 0.64 (95% CI: 0.01–1.00)

MRI, magnetic resonance imaging; DIE, deep infiltrating endometriosis; κ: Cohen’s kappa, κw: kappa with quadratic weights.

## Data Availability

Data are available upon reasonable request.
